# Explorative laparotomy of cecal volvulus in a pediatric patient. A case report and review of literature

**DOI:** 10.1016/j.ijscr.2024.109495

**Published:** 2024-03-11

**Authors:** Lujain Alzahrani, Faisal Joueidi, Fawzy Mohamed Abodahab, Khaled Joueidi, Asim Khan

**Affiliations:** a Department of Pediatric Surgery, Maternity and Children Hospital, Makkah, Saudi Arabia; b College of Medicine, Alfaisal University, Riyadh, Saudi Arabia

**Keywords:** Cecal volvulus, Meckel's diverticulum, Pediatric, Exploratory laparotomy, Case report

## Abstract

**Introduction and importance:**

Cecal volvulus is a rare intestinal pathology that occurs due to abnormal cecum mobility associated with spectrum of complications. It is usually manifested in adults. However, on extremely rare occasions, it occurs in pediatrics. We presented a case of cecal volvulus demonstrating the significance of early diagnosis and treatment to reach successful outcomes.

**Case presentation:**

A 12 year old boy who presented to the emergency department for clinical evaluation for acute abdomen. History and clinical examination was suggestive of acute bowel obstruction. Abdominal x-ray showed a large, distended gas filled viscus with base pointed towards the right lower quadrant. On the bases of radiological investigations, diagnosis of cecal volvolus made. Accordingly, the patient underwent emergency exploratory laparotomy. The post operative course was uneventful and was discharged in stable condition.

**Clinical discussion:**

Cecal volvulus is an extremely rare manifestation of intestinal obstruction and malrotation. The clinical presentation of cecal volvulus depending on the duration and extent of the involvement of cecal malrotation The exact pathogenesis of cecal volvulus is unclear. However the association of the embryological development of the colon, affects the attachment to the posterior parietal peritoneum after ordinary anatomical rotation of 270°. The core-stone management of cecal volvulus is surgical approach.

**Conclusion:**

Cecal volvulus requires a high index of suspicion and delicate care by the pediatric surgeon as it is considered an extremely rare entity in this age group. We highlighted the significance of early diagnosis, surgical treatment and the possibility of developing postoperative complications if left untreated.

## Highlights

• Cecal volvulus is an extremely rare condition in pediatric patients with an estimated incidence of 500 living births.
• Imaging studies done pre-operative can be helpful to diagnosis of cecal volvulus.
• The clinical presentation of cecal volvulus varies depending on the duration and extent of the involvement of cecal malrotation

## 1 Introduction

Cecal volvulus is a rare intestinal malrotation and malfixation anomalies that usually occurs due to abnormal cecum mobility because of cecal mesentery malrotation or improper development of cecal fixation and the ascending colon to the posterior abdominal wall [1,2]. Cecal volvulus is mostly seen in adults. However, it is extremely rarely reported in the pediatric age group with an estimated incidence of 1 in every 500 cases [2,3]. Cecal volvulus is associated with various complications including ischemia, strangulation, perforation, and gangrene [4]. The clinical presentation is highly variable which can delay the diagnosis [1,4]. Usually the diagnosis is made via computed tomography (CT) scan [5]. Treatment options may include cecopexy, cecostomy and appendectomy [2]. We recently treated a 12-year-old boy with the diagnosis of cecal volvulus using explorative laparotomy. The aim of this study is to share our experience to emphasize the importance of early diagnosis and appropriate surgical intervention to establish better outcomes. This case has been reported in line with the SCARE criteria [5].

## 2 Case presentation

**Fig. 1:**
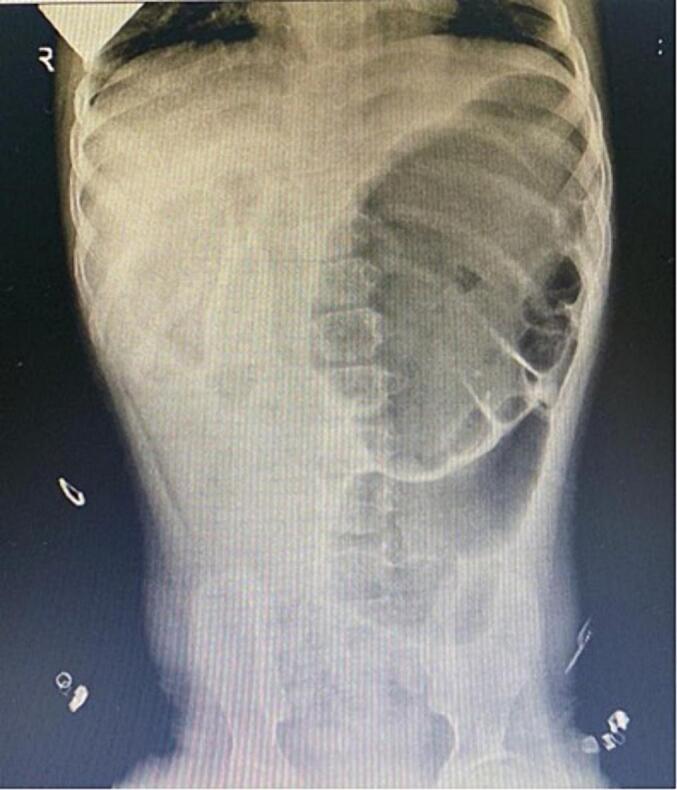
A large, distended gas filled viscus with base pointed towards the right lower quadrant measuring 12.5 cm with small bowel loops in the right upper quadrant.

**Fig. 2:**
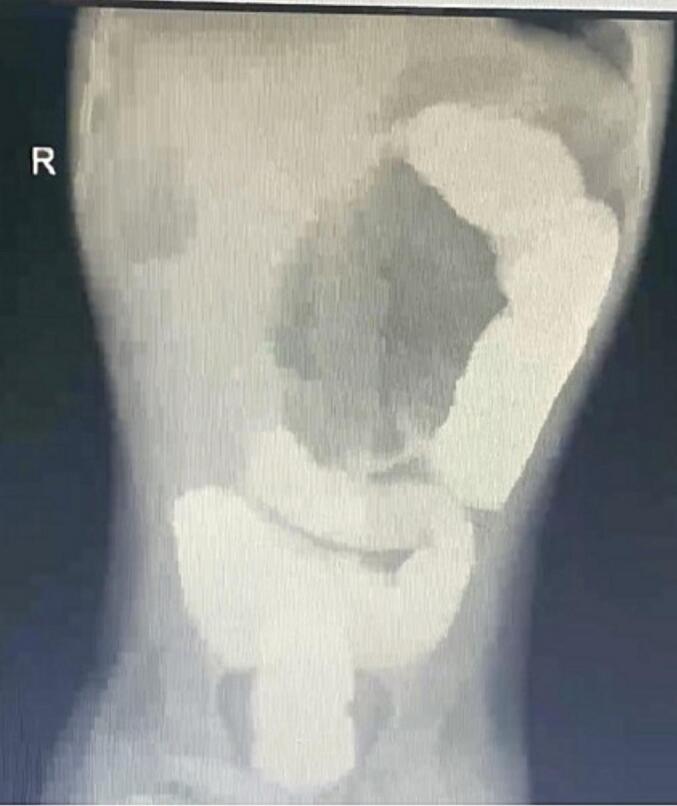
The presence of a stricture/stenotic area at the level of splenic flexure, there was absent opacification of the cecum, ascending colon, and transverse colon.

A 12-year-old boy who is known to have epilepsy and ectodermal dysplasia presented to the emergency department complaining of colicky abdominal pain, vomiting and constipation. The abdominal pain had been ongoing for 4 days prior to hospital presentation. The pain was continuous and progressively worsening. The patient developed vomiting, which started 3 days ago and was yellowish with food content. The patient had history of on-off chronic constipation, passing bowel motion and flatus. Past surgical and family history was unremarkable. Upon examination the patient was conscious, alert and oriented. Abdominal examination revealed a distended abdomen that was soft, lax and not significant tender. Digital rectal examination showed an empty rectum. Laboratory investigations showed WBC: 14.54 10^3 (4.5–13.0 10^3) UL, Hemoglobin 15.4 (13.8–17.2) g/dL, Hematocrit: 46.2 % (40–54 %), sodium 142 (135–145) mmol/L, chloride 109 (96–106) mmol/L, calcium 2.3 (2.13–2.55) mmol/L, PH 7.32 (7.35–7.45), PO2 17 (10.5–13.5), PCO2 35 (35–45). HCO3 18 (22–28), AST 37 (5–32) unit/L, Albumin 39 (30–50) g/L, direct bilirubin 3.3 (0–50) umol/L, Alkaline phosphatase 265 (30–127) g/L, creatinine 66.6 (44–88) umol/L, uric acid 295 (150–420) umol/L. Abdominal x-ray (Anteroposterior and lateral view) showed a large gas filled distended viscus pointing towards the right lower quadrant measuring 12.5 cm with small bowel loops in the right upper quadrant [Fig. 1]. Contrast enema showed the presence of a stricture/stenotic area at the level of splenic flexure and contrast was not passing beyond that stricture, there was absent opacification of the cecum, ascending colon, and transverse colon [Fig. 2]. Therefore, the diagnosis of cecal volvolus was made. Accordingly the patient was resuscitated with intravenous fluid and underwent exploratory laparotomy.

### 2.1 Operative details

**Fig. 3:**
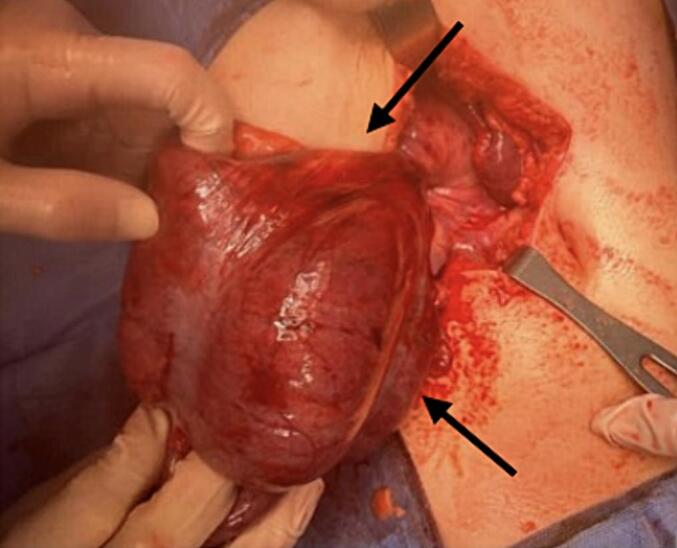
The intraoperative finding showed mobile cecum and part of ascending with hugely dilated and 360° twisted. Though the cecum was dusky in colour but viable.

**Fig. 4:**
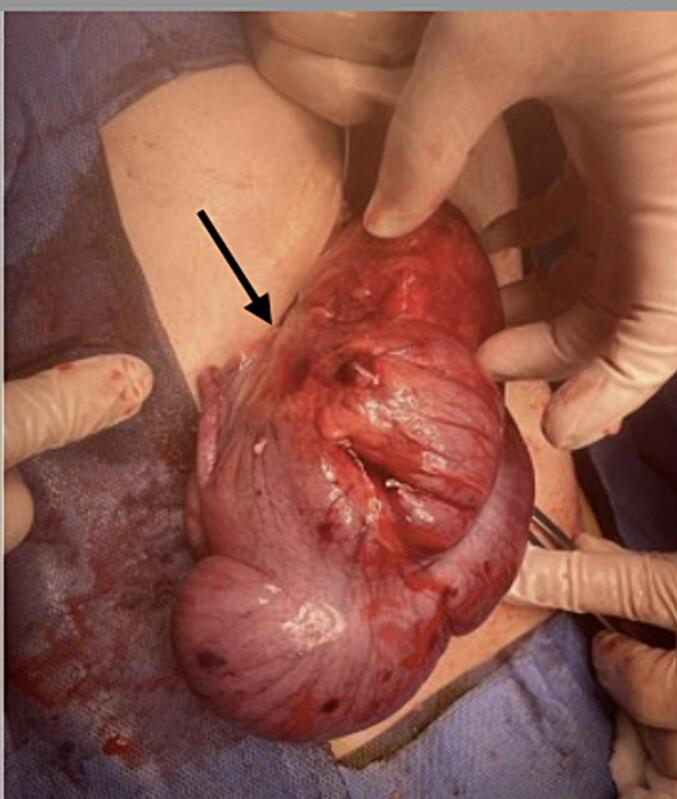
A fixed caecum in the right lower quadrant.

**Fig. 5:**
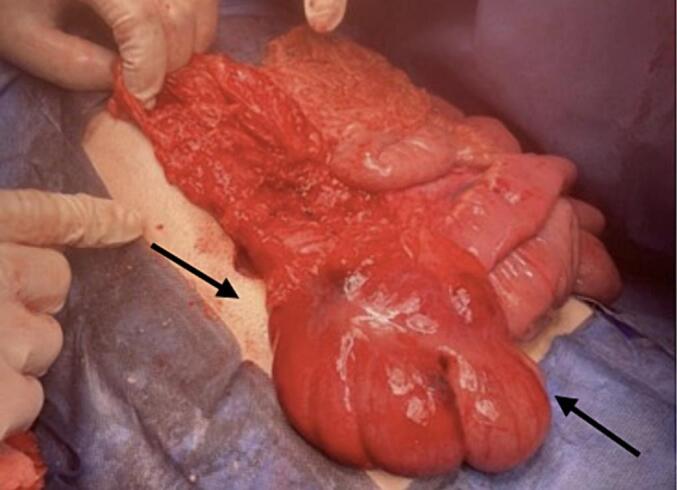
Whole colon distal to the involved segment found to be dilated till the upper part of the rectum.

**Fig. 6:**
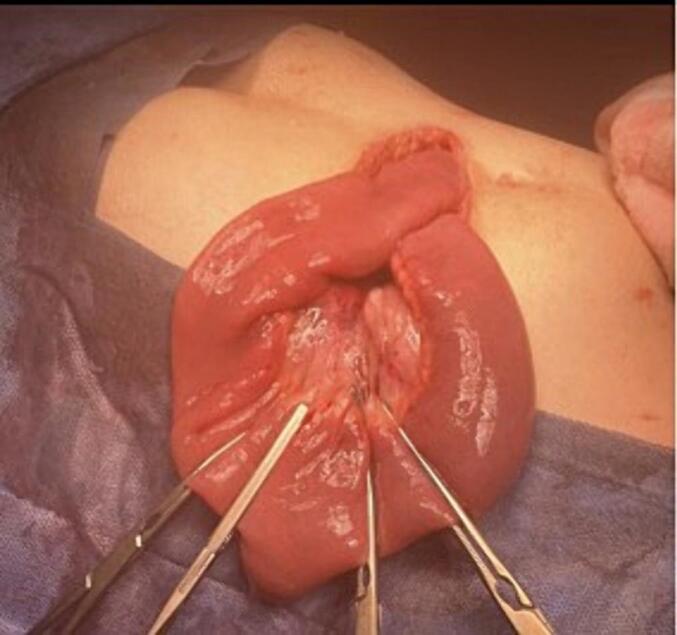
An Incidental finding of wide based Meckel's diverticulum.

A Right upper transverse abdominal incision was made above the umbilicus, revealing intraoperative finding of cecal volvulus. The cecum and part of the ascending colon were mobile, greatly dilated and twisted 360°. Although the cecum appeared dusky in colour but viable. The twisted part of intestine was de-twisted and warm fomentation was applied till it regained normal colour [Fig. 3]. Subsequently, a cecopexy was performed by fixing the caecum in the right lower quadrant and an appendectomy was carried out, the DJ function is at its normal position towards the left side of the spine [Fig. 4]. Additionally the entire colon distal to the affected segment was dilated up to the upper part of the rectum due to chronic constipation. There was incidental discovery of a wide based Meckel's diverticulum and was removed through diverticulectomy [Fig. 5, Fig. 6].

Postoperatively the patient was kept on NPO for three days with IV fluids and intravenous antibiotics. and oral feeding was resumed. Meanwhile, during the hospital stay the patient developed fever on 4th day post operatively due to upper respiratory tract infection. A computed tomography (CT) scan was performed to rule out any intraabdominal collection. Revealing colonic distension with gaseous/ fluid filled and mild dilation of the proximal small bowel. However there were no signs of bowel obstruction or peritoneal collections. Differential diagnosis was suggestive of cecal volvulus, sigmoid volvulus, acute pancreatitis and acute appendicitis. The diagnosis of cecal volvulus was confirmed accordingly the patient was treated with antibiotic for the fever and was discharged in stable condition. During a follow up visit the patient was progressing well and adviced on a bowel management plan and prescribed laxatives for chronic constipation.

## 3 Discussion

Cecal volvulus is an extremely rare form of intestinal obstruction that was first described by Rokitansky in 1841 and is typically caused by abnormal cecal mobility and malfixation [2]. It accounts for 3.4 % of all cases of colonic obstruction and can manifest as an acute fulminating type, acute obstructive type, or chronic recurrent volvulus [2]. The acute fulminating type is an acute onset that can rapidly progress to gangrene with a mortality rate of 65 % of cases [2]. Acute obstructive type is a slow onset type of gangrene that usually manifests as recurrent colicky abdominal pain with right iliac fossa mass and occurs in late settings with a mortality rate reaching 10–25 % of cases [2]. Chronic recurrent volvulus is characterized by recurrent colicky pain and abdominal distention [2]. The clinical presentation depends on the duration and extent of the involvement of cecal malrotation and is highly variable in presentation this includes abdominal pain, vomiting, abdominal distention, and constipation [4,6]. Several factors contribute to the development of cecal volvulus. [7] The most common causes of cecal volvulus are idiopathic colorectal band or congenital colorectal band, extended length of the right colonic mesentery, other etiologies including high residual diet, chronic constipation, Hirschsprung disease, previous abdominal surgery, abdominal masses, stenotic process that diminishes the colon's ability to evacuate, postoperative paralytic ileus, and postoperative adhesions [6,7]. The exact pathogenesis of cecal volvulus is not fully understood [7]. However, studies suggest the association of the embryological development of the colon, the attachment to the posterior parietal peritoneum occurs after ordinary anatomical rotation of 270° [8]. In cecal volvulus, the development of incomplete attachment with the ordinary rotation or stretching of the colon due to over-rotation can result in a mobile cecum. It is generally known that the presence of cecal volvulus occurs due to a mobile cecum lacking a right colon, caecum, terminal ileum, and intestinal attachment to the posterior parietal peritoneum [2]. Despite this possible anatomical predisposition in certain individuals, the exact pathogenesis is likely to be multifactorial [8]. Cecal and ascending colorectal retroperitoneal fixation fails in about 10–15 % of patients [7].

Early diagnosis of cecal volvulus is crucial in early stages as delayed or missed cases can progress to the development of mesenteric ischemia or bowel gangrene [6]. Abdominal radiographic imaging aids in differentiating between sigmoid and cecal volvulus Although, around 30 % of cases may not show in radiographic features [9]. Abdomin-pelvic computed tomography (CT) scan is highly accurate modality in diagnosing cecal volvulus with an estimated success rate of 90 % of cases [10]. Furthermore, it is used as a pre-operative assessment tool which helps in differentiating the location of the torsion and points out other associated abnormalities such as the relationship between the superior mesenteric artery and vein, and underdeveloped or absent uncinate pancreas [7,10]. Young et al. emphasized the importance of distinguishing cecal volvulus from sigmoid volvulus [9]. Barium enema is a confirmatory modality that enables visualization of the distal colon to exclude co-existing abnormalities that can contribute to the development of cecal volvulus [9]. Furthermore, the diagnostic accuracy is reported to be 88 % for acute cecal volvulus [9]. An abdominal X-ray is used to delineate the dilation and the extent of the bowel from the right lower quadrant to the left upper quadrant [10]. However abdominal x-ray is less diagnostic compared to other modalities [10]. Preoperative mortality in cecal volvulus cases carries high risk with an estimated range of 0–40 % depending on the viability of the bowel or gangrene or the type of therapeutic procedure [11].

The optimal approach to manage cecal volvulus remains controversial. Several studies suggested that the safety and efficacy of conservative management depends on the detorsion with or without cecopexy in the absence of intestinal ischemia. However, surgery is widely recognized as the cornerstone treatment for such cases [6,12]. The selection of surgical procedures varies depending on hemodynamic stability and the extent of bowel compromise, this includes caecopexy, and colonoscopy decompression [4,13]. Cecopexy is a safe and minimal invasive procedure that is used to preserve colon function. However, it is associated with a high risk of recurrence in 13 % of cases [13,14]. Laparoscopy has a minimal impact on the management of cecal volvulus [15]. Zouari et al. reported a 12 year old with the diagnosis of cecal volvulus and underwent cecal detorsion, cecopexy and appendectomy [12]. The surgery was complicated with enterocutaneous fistula [12]. Halabi et al. showed that less than 4 % of patients who were treated laparoscopically were young, or had fewer comorbidities [15]. In cases of bowel gangrene surgery offers definitive management and carries the lowest recurrence risk, and reduces the risk of developing septic shock [13,16]. Zineb et al. reported three cases of cecum volvulus case with a picture of acute occlusive syndrome. Accordingly two patients underwent surgical resection with anastomosis and one underwent caecopexy. Postoperatively the patients tolerated the procedure [17].

## 4 Conclusion

Cecal volvulus is an extremely rare anomaly that has been reported in medical literature. This particular case highlights the rare existence of cecal volvulus in pediatric age group. However in cases of chronic constipation or sigmoid volvulus it should be considered as differential diagnosis. Prompt diagnostic intervention and surgically treating young patients with signs and symptoms of abdominal obstruction is crucial in preventing potential mortality and avoiding long-term complications.

## Patient consent

Consent to publish the case series was obtained. This report does not contain any personal information that could lead to the identification of the patient.

## Methods

The work has been reported in line with the SCARE criteria.

Sohrabi C, Mathew G, Maria N, Kerwan A, Franchi T, Agha RA. The SCARE 2023 guideline: updating consensus Surgical CAse REport (SCARE) guidelines. Int J Surg Lond Engl. 2023;109(5):1136.

## Ethical approval

This is a case report and according DHHS is a medical/educational activity that does not meet the DHHS definition of “research”.

## Funding

There were no funding sources.

## Author contribution

F. Joueidi, F. Abodahab and K. Joueidi participated in writing the background with significance, case presentations, discussion, and conclusion, read and approved the final manuscript.

L. Alzahrani, F. Joueidi helped draft the manuscript, read, and agree on the final manuscript, and formatted the manuscript according to the journal's guidelines.

A. Khan, L. Alzahrani, supervising the overall research, preparing the manuscript reading, and approving the final manuscript.

## Guarantor

Asim Khan accepts full responsibility for the work and/or the conducted of the study, had access to the data and controlled the decision to publish.

## Declaration of competing interest

The authors declare that they have no known competing financial interests or personal relationships that could have appeared to influence the work reported in this paper.
